# TOM-1/tomosyn acts with the UNC-6/netrin receptor UNC-5 to inhibit growth cone protrusion in *Caenorhabditis elegans*

**DOI:** 10.1242/dev.201031

**Published:** 2023-04-04

**Authors:** Snehal S. Mahadik, Erik A. Lundquist

**Affiliations:** Department of Molecular Biosciences, The University of Kansas, 1200 Sunnyside Avenue, 5049 Haworth Hall, Lawrence, KS 66045, USA

**Keywords:** Axon guidance, Growth cone, TOM-1/tomosyn, UNC-5, UNC-6/netrin

## Abstract

In the polarity/protrusion model of growth cone repulsion from UNC-6/netrin, UNC-6 first polarizes the growth cone of the VD motor neuron axon via the UNC-5 receptor, and then regulates protrusion asymmetrically across the growth cone based on this polarity. UNC-6 stimulates protrusion dorsally through the UNC-40/DCC receptor, and inhibits protrusion ventrally through UNC-5, resulting in net dorsal growth. Previous studies showed that UNC-5 inhibits growth cone protrusion via the flavin monooxygenases and potential destabilization of F-actin, and via UNC-33/CRMP and restriction of microtubule plus-end entry into the growth cone. We show that UNC-5 inhibits protrusion through a third mechanism involving TOM-1/tomosyn. A short isoform of TOM-1 inhibited protrusion downstream of UNC-5, and a long isoform had a pro-protrusive role. TOM-1/tomosyn inhibits formation of the SNARE complex. We show that UNC-64/syntaxin is required for growth cone protrusion, consistent with a role of TOM-1 in inhibiting vesicle fusion. Our results are consistent with a model whereby UNC-5 utilizes TOM-1 to inhibit vesicle fusion, resulting in inhibited growth cone protrusion, possibly by preventing the growth cone plasma membrane addition required for protrusion.

## Teaser

**Summary:** The short isoform of TOM-1/tomosyn inhibits growth cone protrusion downstream of the netrin receptor UNC-5 during growth cone outgrowth, whereas long isoforms have a pro-protrusive role.

## INTRODUCTION

Guided extensions of axons and dendrites play a crucial role in the development of neural circuits and networks. Growth cones present at the growing tip of the neurite guide the outgrowth of the neurite. Growth cones are dynamic structures comprising a branched actin lamellipodial body and filopodial protrusions consisting of F-actin bundles (Gallo and Letourneau, 1999; Gallo and Letourneau, 2004; Zhou and Cohan, 2004; Pak et al., 2008; Lowery and Van Vactor, 2009). Extracellular guidance cues are detected by growth cones via receptors present on the plasma membrane, which orchestrate a series of intracellular events that guide the growth cone in the proper direction (Tessier-Lavigne and Goodman, 1996; Gallo and Letourneau, 1999; Gallo and Letourneau, 2004; Lowery and Van Vactor, 2009), including actin cytoskeletal dynamics, microtubule delivery of vesicles and cytoskeletal regulators, and addition of plasma membrane via exocytosis.

In *Caenorhabditis elegans* and vertebrates, UNC-6/netrin is a bifunctional, conserved, secreted laminin-like guidance cue, which directs dorsal-ventral axon guidance by utilizing its receptors UNC-40/DCC and UNC-5 (Hedgecock et al., 1990; Ishii et al., 1992; Chan et al., 1996; Norris and Lundquist, 2011). VD motor neuron cell bodies reside in the ventral nerve cord and send processes anteriorly in the ventral nerve cord, which then turn dorsally and migrate to the dorsal nerve cord, forming an axon commissure. Dorsal commissural growth of the VD growth cone is dependent upon UNC-6 in the ventral nerve cord (i.e. VD growth cones migrate away from UNC-6/netrin, classically called repulsion) (Wadsworth et al., 1996; Wadsworth, 2002; Norris and Lundquist, 2011). As the VD growth cone migrates dorsally, it extends dynamic filopodial protrusions biased to the dorsal direction of growth (Knobel et al., 1999; Norris and Lundquist, 2011).

Classically, it was thought that UNC-6/Netrin forms a ventral-to-dorsal gradient that growth cones dynamically sense (Tessier-Lavigne and Goodman, 1996; Boyer and Gupton, 2018), with growth up (attraction) or down (repulsion) the gradient involving the UNC-40 receptor and UNC-5, respectively. Recent studies in vertebrate spinal cord suggest that gradients are not involved and that netrin 1 acts in a short-range, haptotactic mechanism in spinal cord commissural guidance (Dominici et al., 2017; Varadarajan and Butler, 2017; Yamauchi et al., 2017; Morales, 2018).

In *C. elegans*, *in vivo* imaging studies of VD growth cones in wild type and *unc-6* signaling mutants also do not support the gradient model. Instead, they indicate that UNC-6 first polarizes the growth cone via the UNC-5 receptor, and then regulates growth cone protrusion based on this polarity (Norris and Lundquist, 2011; Norris et al., 2014; Gujar et al., 2018). UNC-5 inhibits protrusion ventrally, and UNC-40 stimulates protrusion dorsally, resulting in net dorsal growth. Thus, both UNC-5 and UNC-40 receptors act in the same growth cone to promote growth away from UNC-6 by balancing protrusion across the growth cone. This polarity/protrusion model is a new paradigm for understanding the role of UNC-6/netrin in axon guidance. The statistically oriented asymmetric localization (SOAL) model involving UNC-40 acts similarly to direct growth cone growth toward UNC-6 (Kulkarni et al., 2013; Yang et al., 2014; Limerick et al., 2018).

Central to the polarity/protrusion model is the finding that UNC-5 polarizes the growth cone and then inhibits growth cone lamellipodial and filopodial protrusion based on this polarity. UNC-40 stimulates protrusion at the dorsal growing tip, and UNC-5 inhibits protrusion ventrally and laterally (Norris and Lundquist, 2011; Norris et al., 2014; Gujar et al., 2018). Previous studies indicate that UNC-5 inhibits VD growth cone protrusion through two mechanisms. The flavin monooxygenases (FMOs) act downstream of UNC-5 to inhibit protrusion, possibly by destabilizing F-actin, similar to the FMO protein MICAL (Gujar et al., 2017). UNC-33/CRMP acts downstream of UNC-5 and inhibits protrusion by restricting microtubule plus-end entry into the growth cone (Gujar et al., 2018). Microtubules are pro-protrusive in the VD growth cones (Gujar et al., 2018, 2019), possibly acting by delivering vesicles and cytoskeletal regulators, such as Arp2/3 and Enabled (Norris et al., 2009).

Here, a potential third pathway downstream of UNC-5 in inhibiting protrusion is explored, involving regulation of vesicle exocytosis and addition of plasma membrane to the growth cone. Vesicle exocytosis is required for growth cone membrane extensions by fusion of plasmalemmal precursor vesicles at the plasma membrane of growth cones and is closely tied to cytoskeletal dynamics (Futerman and Banker, 1996; Hausott and Klimaschewski, 2016; Nozumi and Igarashi, 2018), and a balance of endocytosis and exocytosis is involved in growth cone guidance (Tojima et al., 2014; Tojima and Kamiguchi, 2015). The axon guidance cue reelin controls fusion of VAMP7-positive vesicles in regenerating dorsal root ganglion neurons (Jausoro and Marzolo, 2021). Furthermore, synaptic-like vesicles in the growth cone are required for pioneer axon navigation in zebrafish (Nichols and Smith, 2019). In *C. elegans*, the RAB-3 GTP exchange factor AEX-3 controls pioneer axon guidance (Bhat and Hutter, 2016). Studies on rat cultured hippocampal neurons suggest that exocytosis is restricted to the distal, dynamic region of growth cones, which leads to membrane addition and extension (Sakisaka et al., 2004).

Tomosyn (also known as syntaxin-binding protein 5) was identified as a syntaxin-interacting molecule that was shown to inhibit interaction of the T-SNARE syntaxin with the V-SNARE synaptobrevin, thus blocking formation of the SNARE complex and preventing vesicle fusion (Fujita et al., 1998). Tomosyn has been well-characterized in relation to inhibition of synaptic vesicle fusion and dense core vesicle fusion in the nervous system (Fujita et al., 1998; Hatsuzawa et al., 2003; Widberg et al., 2003; Pobbati et al., 2004; McEwen et al., 2006; Takamori et al., 2006; Gladycheva et al., 2007), including in *C. elegans* (Dybbs et al., 2005; Gracheva et al., 2006; McEwen et al., 2006; Gracheva et al., 2010; Burdina et al., 2011). Tomosyn also regulates growth cone morphology in cultured rat hippocampal neurons. Vesicle fusion is inhibited in the proximal ‘palm’ of the growth cone owing to the action of tomosyn in this region (Sakisaka et al., 2004). When growth cones undergo collapse, tomosyn relocalizes around the perimeter of the growth cone (Sakisaka et al., 2004).

In this work, the role of TOM-1 in VD growth cone morphology is explored as well as its interaction with UNC-5 in inhibiting VD growth cone protrusion. Although complete loss of TOM-1 had little effect on VD growth cone morphology, it did suppress growth cone inhibition of protrusion driven by activated MYR::UNC-5. This suggests that TOM-1 is required for MYR::UNC-5 to inhibit growth cone protrusion and that TOM-1 might act downstream of UNC-5 in this process. *tom-1* encodes long (TOM-1L) and short (TOM-1S) isoforms. The long isoform contains N-terminal WD40 repeats and a C-terminal R-SNARE domain that interacts with syntaxin, whereas the short isoform only encodes the C-terminal R-SNARE domain and lacks the WD40 repeats. A mutation that eliminates only the long isoform but not the short isoform resulted in small, less-protrusive VD growth cones. Furthermore, *tom-1L* mutation suppressed the excess VD growth cone protrusion observed in *unc-5* loss-of-function mutants. These results suggest that the TOM-1L and TOM-1S isoforms have opposing functions, with TOM-1S acting in an anti-protrusive manner and TOM-1L being pro-protrusive. Mutation of *tom-1S* without affecting *tom-1L* led to a phenotype resembling that of complete loss of *tom-1* (no effect on VD growth cone alone but suppression of *myr::unc-5*). Furthermore, transgenic expression of *tom-1S* resulted in reduced VD growth cone protrusion. Taken together, these data suggest that in VD growth cones *tom-1S* is the active isoform in inhibiting growth cone protrusion and possibly vesicle fusion, whereas *tom-1L* has a pro-protrusive role. *unc-64* encodes the T-SNARE syntaxin. A hypomorphic *unc-64* mutant had small, less-protrusive VD growth cones and suppressed the excess protrusion of *unc-5* loss-of-function mutants. In sum, these results are consistent with a model wherein UNC-5 engages TOM-1S to inhibit vesicle fusion and thus inhibit growth cone protrusion. Additionally, *tom-1L*, *tom-1S* and *unc-64* were each required for VD growth cone polarity of protrusion, suggesting that regulated vesicle fusion is necessary to establish and/or maintain VD growth cone polarity.

## RESULTS

### *tom-1* encodes long and short isoforms

**Fig. 1.**
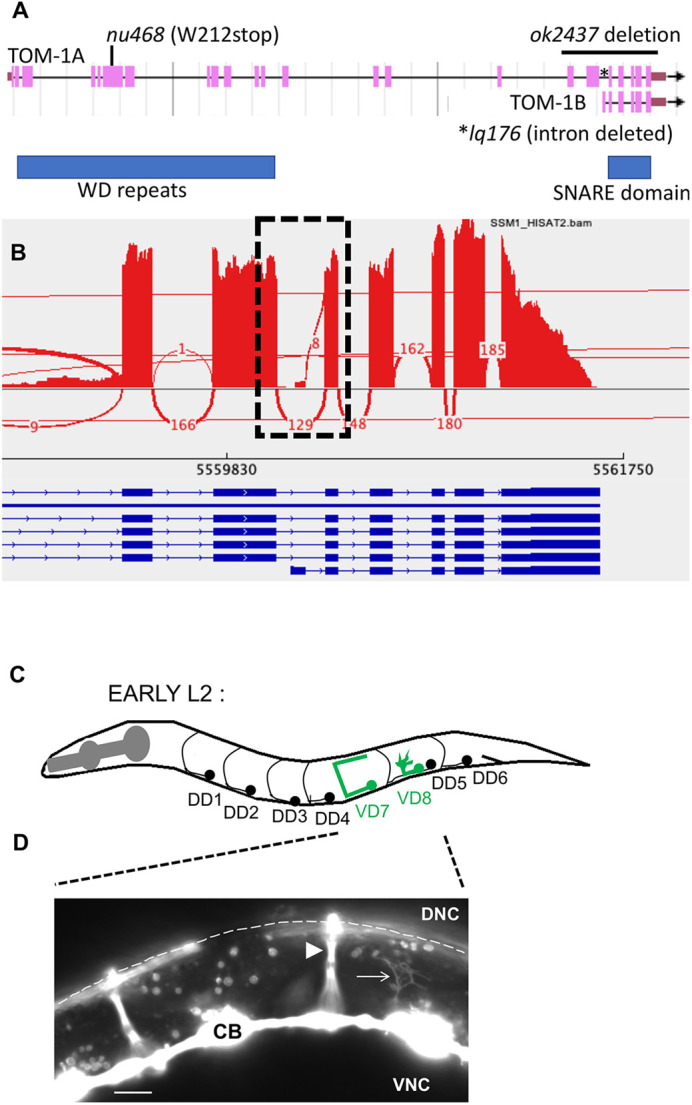
***tom-1* gene structure and VD growth cones.** (A) *tom-1* gene structure (from WormBase WS284). *tom-1A* is representative of the six long isoforms, and *tom-1B* is the short isoform. Regions encoding the WD40 repeats, and the SNARE domain are indicated by blue boxes. Alleles and location of their molecular lesions producing multiple isoforms are indicated. *tom-1(nu468)* results in a premature stop codon predicted to affect all long isoforms but not *tom-1B* short. *tom-1(ok2437*) is a 2391-bp deletion that removes all of exons 17 to 23 and is predicted to affect all isoforms of *tom-1*. *tom-1(lq176)* is a precise deletion of intron 18 of *tom-1A*, leaving the coding potential for *tom-1A* unchanged but deleting the first exon of *tom-1B*. (B) Sashimi plot from the Integrated Genome Viewer of splice junctions in RNA-seq reads of *tom-1.* RNA-seq was conducted on mixed stage N2 animals, reads aligned using HISAT2, and analyzed with the Integrated Genome Viewer. Gene structure is shown below in blue, and splice junctions with their abundance in the RNA-seq reads are indicated. The dashed box shows splices at intron 18, of which 8/137 include the *tom-1B* first exon. (C) Diagram of an early L2 larva of a *C. elegans* hermaphrodite highlighting the structure and position of the DD motor neurons and axons (black), and VD7 and VD8 (green). Dorsal is up, and anterior is left. A growth cone is represented on the VD8 axon as it extends dorsally. (D) A fluorescence micrograph of an early L2 animal with *unc-25::gfp* expression in the VD and DD neurons. Dorsal is up, and anterior is left. Arrowhead indicates a DD commissural axon, and arrow points to a VD growth cone as it extends dorsally. The dashed line indicates the position of the dorsal nerve cord. CB, cell body; DNC, dorsal nerve cord; VNC, ventral nerve cord. Scale bar: 5 μm. A region of this image is shown in Figs 4D, 7D and 9D as a representative image of a wild-type growth cone.

The *C. elegans* genome encodes a single tomosyn gene, *tom-1*, which was identified in a forward genetic screen for enhancers of acetylcholine secretion (Dybbs et al., 2005). TOM-1 is an ortholog of mammalian tomosyn and shares a significant sequence similarity with mammalian tomosyn 1 (Stxbp5) and tomosyn 2 (Stxbp5l) (Gracheva et al., 2006). Through alternative 5′ end usage and alternative splicing, *tom-1* produces at least seven isoforms (WormBase): six long isoforms, including *tom-1A*, and a single short isoform encoded by *tom-1B* (Fig. 1A), produced by alternative 5′ exon located in an intron of *tom-1A*. RNA sequencing (RNA-seq) on three independent replicates of mixed-stage, wild-type animals revealed that the *tom-1B* 5′ exon and splice occurred in 8/137, 9/151 and 4/109, or ∼5%, of splicing events involving this intron (Fig. 1B). This is likely to be an underestimate owing to the location of this small exon at the 5′ end on the transcript and it not being completely represented during RNA-seq library construction. Long isoforms of *tom-1* encode conserved WD40 repeats in the N terminus, and a conserved R-SNARE-like domain at the C terminus, through which tomosyn interacts with the SNARE complex (Fig. 1A) (Gracheva et al., 2006). The short *tom-1B* isoform lacks the N-terminal WD40 repeats and encodes only the C-terminal R-SNARE domain.

*tom-1(ok2437)* is a 2391 bp deletion that removes 3′ exons predicted to affect the short and long isoforms of *tom-1* (Fig. 1A), and *tom-1(nu468)* introduces a premature stop codon at tryptophan at 212 (Dybbs et al., 2005) and is predicted to affect only the long isoforms and not the *tom-1B* short isoform (Fig. 1A).

### TOM-1 regulates VD growth cone protrusion and polarity

**Fig. 2.**
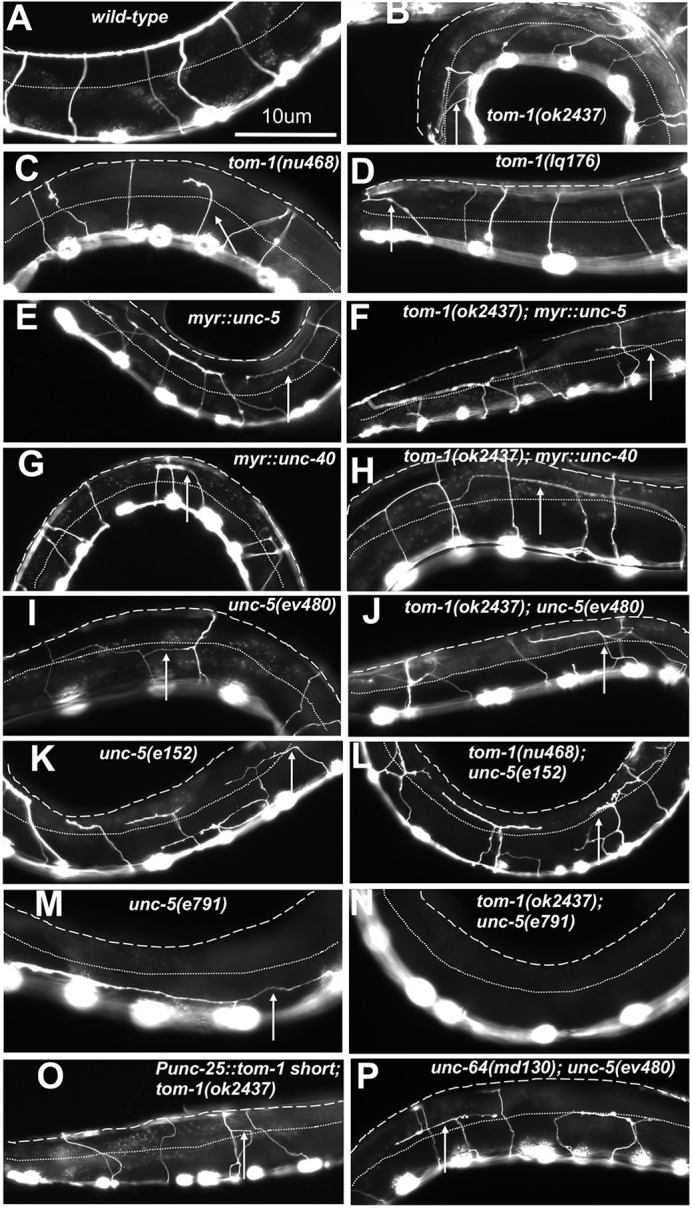
**VD/DD axon guidance defects.** (A-P) Fluorescence micrographs of the *Punc-25::gfp* transgene *juIs76* expressed in the VD/DD neurons of L4 animals. Dorsal is up and anterior left. The approximate lateral midline is indicated with a dotted white line, and the dorsal nerve cord by a dashed white line. White arrows indicate axon guidance defects in each genotype. Scale bar:10 μm. Genotypes are indicated in each figure panel.

**Fig. 3.**
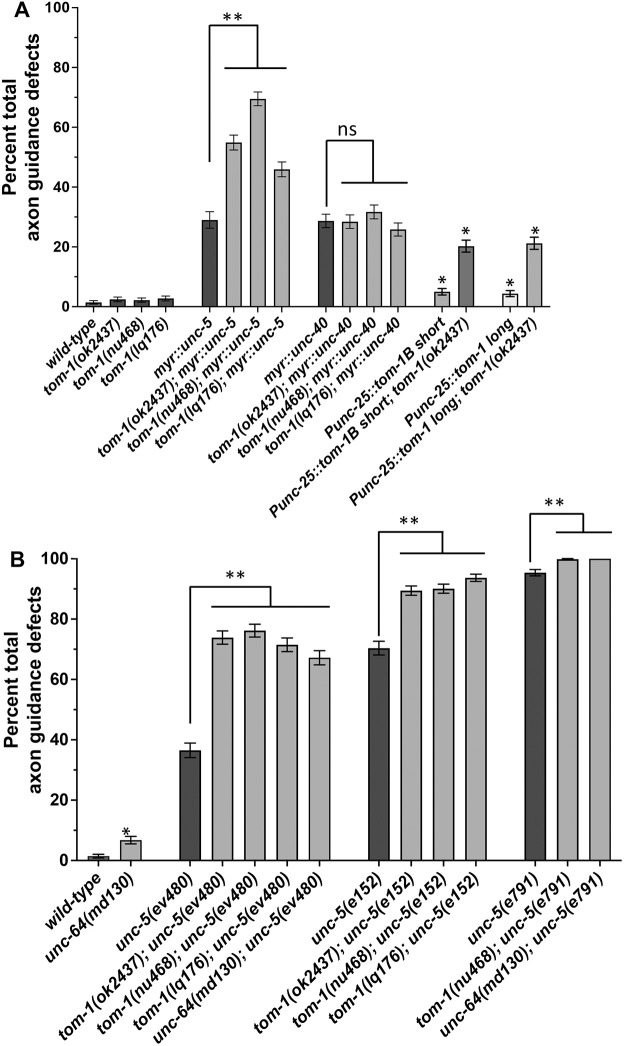
**Quantification of VD/DD axon guidance defects.** (A,B) Total axon guidance defects were quantified in the VD/DD neurons of L4 animals, as described in Materials and Methods. One-hundred animals of each genotype were analyzed. In wild type, each animal had on average 16 VD/DD commissural processes apparent (1600 processes total were scored for each genotype). Genotypes are listed on the *x*-axis, and the percentage of animals exhibiting axon guidance defects on the *y*-axis. Error bars represent 2× standard error of proportion. Significance of difference was determined using Fisher's exact test. Single asterisks indicate significance compared with wild type; double asterisks indicate significance of single mutants alone compared with the predicted additive effect of double mutants calculated by the formula p1+p2 – (p1p2). ns, not significant.

In the early L2 larval stage, axons of the VD neurons begin their ventral-to-dorsal commissural growth (Fig. 2A), with a visible growth cone at the tip of the extending commissural VD axons (Fig. 1C,D). DD axons extend earlier, in late embryogenesis. *tom-1(nu468)* and *tom-1(ok2437)* mutants each displayed increased levels of axon guidance defects compared with wild type. However, these increases were not statistically significant (Fig. 2B-D; Fig. 3A).

**Fig. 4.**
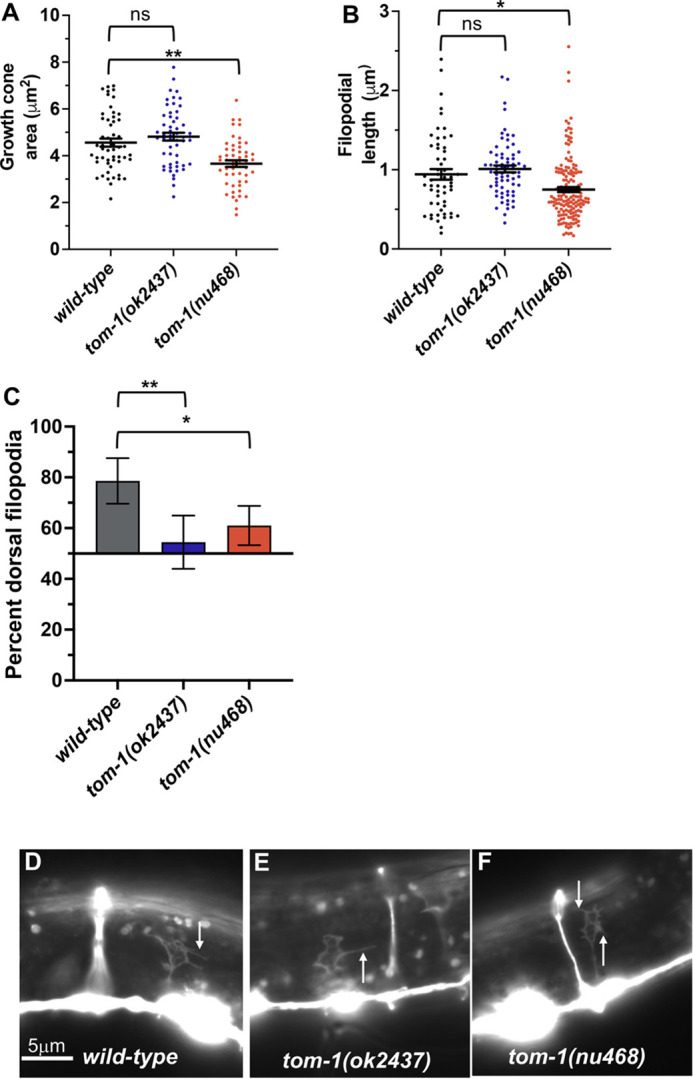
**Growth cone analysis in *tom-1(ok2437)* and *tom-1(nu468).*** At least 50 growth cones were scored for each genotype. In the graphs, each point represents a measurement of a single growth cone or filopodium. (A,B) Quantification of VD growth cone area (A) and filopodial length (B) (see Materials and Methods). Error bars indicate s.e.m. Two-sided *t-*tests with unequal variance were used to determine significant differences between wild type and mutants. *P<0.05, **P<0.001. (C) Percentage of dorsally directed filopodial protrusions in VD growth cones of different genotypes (see Materials and Methods). The *x*-axis is set at 50%, such that bars extending above the *x*-axis represents above 50%, and bars that extend below represent below 50%. In wild type, the majority of filopodia (78%) extended from the dorsal half of the growth cone. Significant differences between wild type and mutants were determined by Fisher's exact test. Error bars represent 2× standard error of proportion. *P<0.05, **P<0.001. (D-F) Fluorescence micrographs of wild-type and mutant VD growth cones expressing *Punc-25::gfp*. The image in D is a cropped version of the wild-type growth cone shown in Fig. 1D and is also shown in Figs 7D and 9D as a representative image of a wild-type growth cone. Arrows point to filopodial protrusions. Dorsal is up; anterior is left. Scale bar: 5 μm.

Growth cone morphology of VD axons in *tom-1* mutants was analyzed as previously described (Norris and Lundquist, 2011; Norris et al., 2014; Mahadik and Lundquist, 2022). Wild-type VD growth cones displayed an average area of 4.6 μm^2^ (Fig. 4A,D), and filopodial protrusions with an average length of 0.9 μm (Fig. 4B,D). VD growth cones are polarized, with filopodial protrusions biased to the dorsal aspect of the growth cone, the direction of growth (Fig. 4C,D). *tom-1(ok2437)*, an allele affecting both long and short isoforms, displayed a slight but not statistically significant increase in VD growth cone area and filopodial length (Fig. 4A,B,E). The long isoform-specific *tom-1(nu468)* mutant VD growth cones displayed significantly reduced growth cone area and filopodial length (Fig. 4A,B,F). These results suggest that *tom-1L* isoforms might have a pro-protrusive role in the growth cone.

Dorsal polarity of growth cone protrusion was significantly reduced in both *tom-1(ok2437)* and *tom-1(nu468)* mutants (Fig. 4C,E,F). These data suggest that TOM-1L isoforms are required to maintain dorsal polarity of filopodial protrusions. The long isoform-specific *tom-1(nu468)* mutant displayed reduced growth cone area and shorter filopodial protrusions, whereas the *tom-1(ok2437)* mutant, in which both long and short isoforms are affected, did not. This suggests that the long and short isoforms of TOM-1 might have distinct roles in regulation of VD growth cone morphology.

### TOM-1 is required for the effects of activated MYR::UNC-5 on VD growth cone morphology

**Fig. 5.**
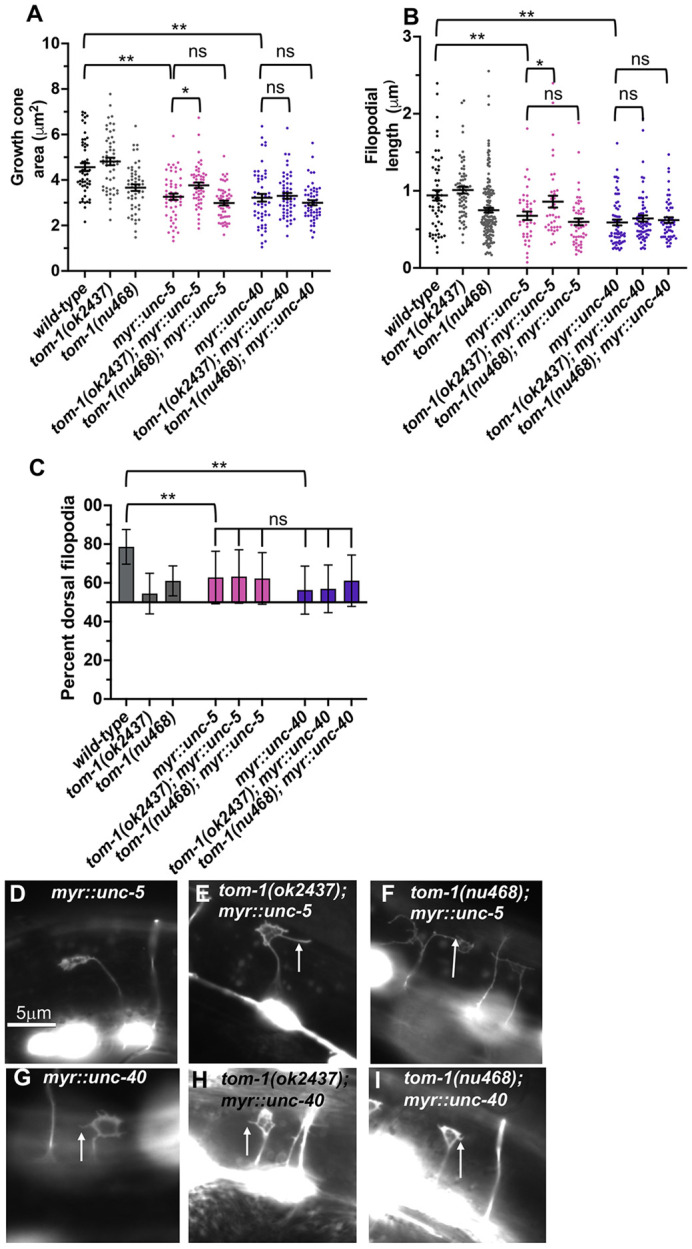
**Growth cone analysis of double mutants of *tom-1(ok2437)* and *tom-1(nu468)* with *myr::unc-5* and *myr::unc-40*.** At least 50 growth cones of each genotype were analyzed. (A,B) Quantification of VD growth cone area and filopodial length as described in Fig. 4. Statistical comparisons between genotypes shown on other figures are not shown again here. Brackets indicate genotypes analyzed for significance. (C) Percentage of dorsally directed filopodial protrusions in VD growth cones of different genotypes as described in Fig. 4. *myr::unc-5* and *myr::unc-40* significantly perturbed growth cone polarity (brackets), but no *tom-1* double mutant showed any significant difference (ns, not significant). (D-I) Fluorescence micrographs of mutant VD growth cones of the indicated genotypes expressing *Punc-25::gfp*. The image of the *myr::unc-5* growth cone in D is identical to the representative *myr::unc-5* growth cone image shown in Fig. 7F. Arrows point to filopodial protrusions. Dorsal is up; anterior is left. Scale bar: 5 μm.

Previous work showed that UNC-6/netrin signaling regulates VD growth cone protrusion, including growth cone area, filopodial length, and polarity (Norris and Lundquist, 2011; Norris et al., 2014; Gujar et al., 2017). The UNC-6 receptor UNC-5 normally inhibits growth cone protrusion. Constitutively active MYR::UNC-5 expression in VD growth cones resulted in small growth cones with few and shortened filopodial protrusions (Fig. 5A,B,D). UNC-5 can act as a heterodimer with the UNC-6 receptor UNC-40, and constitutively activated MYR::UNC-40 also resulted in reduced growth cone protrusion (Fig. 5A,B,G), in agreement with previous studies (Gitai et al., 2003; Norris and Lundquist, 2011; Norris et al., 2014; Gujar et al., 2017).

*tom-1(ok2437)*, which affects both long and short isoforms, significantly suppressed the effects of *myr::unc-5. tom-1(ok2437); myr::unc-5* growth cones were larger with longer filopodia compared with *myr::unc-5* alone (Fig. 5A,B,D,E). In contrast, *tom-1(ok2437)* had no effect on growth cone area or filopodial reduction caused by *myr::unc-40* (Fig. 5A,B,G,H)*.* Although loss of *tom-1* alone caused no VD growth cone phenotype, a role of TOM-1 in inhibiting growth cone protrusion was revealed in these studies using the *myr::unc-5* sensitized background. These results are consistent with TOM-1 acting downstream of UNC-5 to inhibit growth cone protrusion. As *myr::unc-40* was unaffected, TOM-1 might act specifically downstream of UNC-5 and not UNC-5::UNC-40 heterodimers.

*tom-1(nu468)*, which affects only the *tom-1L* isoforms and not the *tom-1S* isoform, did not suppress the inhibition of growth cone area and filopodial length caused by *myr::unc-5* or *myr::unc-40* (Fig. 5A,B,D,F,G,I). This suggests that the TOM-1L is not required to inhibit growth cone protrusion. Alone, *tom-1(nu468)* mutants displayed reduced VD growth cone protrusion (Fig. 4), suggesting a pro-protrusive role of the long isoform. Thus, TOM-1 short and long isoforms might have opposing roles, with TOM-1 short normally inhibiting protrusion and TOM-1 long normally stimulating protrusion. These data also suggest that the inhibitory function of the short isoform of TOM-1 does not require the long isoform. *tom-1(ok2437)*, *tom-1(nu468)*, *myr::unc-5* and *myr::unc-40* each affected growth cone polarity, and polarity was unchanged in double mutants (Fig. 5C). This consistent with both *tom-1* mutants alone affecting polarity of protrusion (Fig. 4C).

*tom-1; myr::unc-5* double mutants showed a synergistic increase in VD/DD axon guidance defects, but *tom-1; myr::unc-40* double mutants did not (Figs 2E-H, 3), consistent with TOM-1 specifically interacting with UNC-5. It is noted that, despite restoration of MYR::UNC-5 growth cone protrusion by *tom-1(ok2437)*, axon guidance defects were increased. This suggests that growth cone protrusion is not the only aspect of growth cone dynamics during axon guidance affected by these molecules.

### TOM-1L isoforms are required for excess growth cone protrusion in *unc-5* loss-of-function mutants

**Fig. 6.**
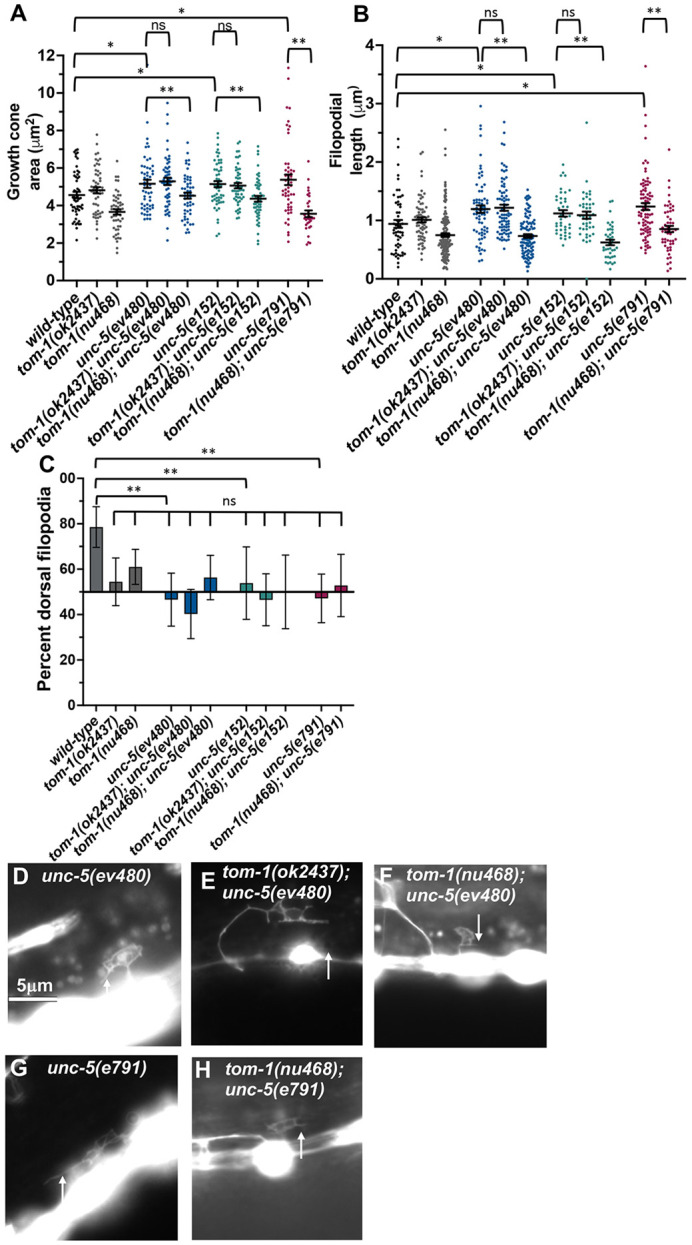
**Growth cone analysis of double mutants of *tom-1(ok2437)* and *tom-1(nu468)* with *unc-5*.** At least 30 growth cones of each genotype were analyzed. (A,B) Quantification of VD growth cone area and filopodial length as described in Fig. 4. Statistical comparisons between genotypes shown on other figures are not shown again here. Brackets indicate comparisons between genotypes. (C) Percentage of dorsally directed filopodial protrusions as described in Fig. 4. *unc-5* mutants exhibited significantly perturbed growth cone polarity (brackets), but no *tom-1* double mutant showed any significant difference (ns, not significant). (D-H) Fluorescence micrographs of mutant VD growth cones of the indicated genotypes expressing *Punc-25::gfp*. Arrows point to filopodial protrusions. Dorsal is up; anterior is left. Scale bar: 5 μm.

UNC-5 has been previously shown to inhibit VD growth cone protrusion (Norris and Lundquist, 2011; Norris et al., 2014). As previously reported, *unc-5(e791)* null mutants displayed increased growth cone area and filopodial length (Fig. 6A,B,G). *unc-5(ev480)* and *unc-5(e152)* are hypomorphic alleles that retain some UNC-5 function (Merz et al., 2001; Killeen et al., 2002; Mahadik and Lundquist, 2022). However, *unc-5(ev480)* and *unc-5(e152)* displayed VD growth cone morphology similar to the *unc-5(e791)* null (Fig. 6A,B,D,G). VD growth cones of *unc-5* mutants also lacked the dorsal bias of growth cone protrusions (Fig. 6C). *unc-5(e791)* mutants displayed a nearly complete failure of VD/DD axons to reach the dorsal nerve cord, whereas *unc-5(ev480)* and *unc-5(e152)* displayed weaker axon guidance defects owing to their hypomorphic nature (Fig. 3).

VD growth cones of double mutants of the *tom-1(ok2437)* null allele and the *unc-5(e791)* null allele could not be scored because none emerged from the ventral nerve cord. There was a resulting complete failure of VD or DD axons extending out of the ventral nerve cord (Fig. 2M,N). VD growth cones of *tom-1(ok2437)* with hypomorphic *unc-5(e152)* and *unc-5(ev480)* were apparent, and they resembled the growth cones of *unc-5* hypomorphs alone, with increased growth cone area and filopodial length compared with wild type (Fig. 6A,B,E). Also, synergistic enhancement of VD/DD axon guidance defects compared with *unc-5(ev480, e152)* alone were observed (Figs 2I,J, and 3). Thus, *tom-1(ok2437)* did not alter the growth cone phenotypes of *unc-*5 hypomorphs, and in fact enhanced VD/DD axon guidance defects.

In contrast, the long isoform-specific *tom-1(nu468)* mutant elicited significantly suppressed growth cone area and filopodial length in all *unc-5* mutants (Fig. 6A,B,F,H). Despite suppression of excessive growth cone protrusion, there was a synergistic enhancement in VD/DD axon guidance defects of these double mutants (Figs 2K,L and 3). This suggests that these genes might have additional roles in growth cone outgrowth not described here. VD growth cones in all *tom-1; unc-5* mutants in which they were apparent were unpolarized, similar to all of the single mutants alone (Fig. 6C).

These studies indicate that the TOM-1 long isoforms are in part necessary for excessive growth cone protrusion in *unc-5* mutants, consistent with a pro-protrusive role of the TOM-1 long isoforms. This, combined with an allele of *tom-1* affecting both long and short isoforms suppressing activated MYR::UNC-5, suggests that the TOM-1 long and short isoforms have opposing roles in regulating growth cone protrusion, with TOM-1L isoforms being pro-protrusive and TOM-1S inhibiting protrusion.

### A *tom-1B* short (*tom-1S*) isoform-specific mutant resembles an allele of *tom-1* affecting both long and short isoforms

The *tom-1S* isoform is produced by an alternative 5′ exon located in an intronic region of the long isoform (Fig. 1A). We used Cas9 genome editing to precisely delete the intron containing the *tom-1B* 5′ exon, effectively fusing together exons 18 and 19 without affecting coding capacity of these exons (Fig. 1). This *tom-1(lq176)* allele is predicted to encode the long isoform of TOM-1.

**Fig. 7.**
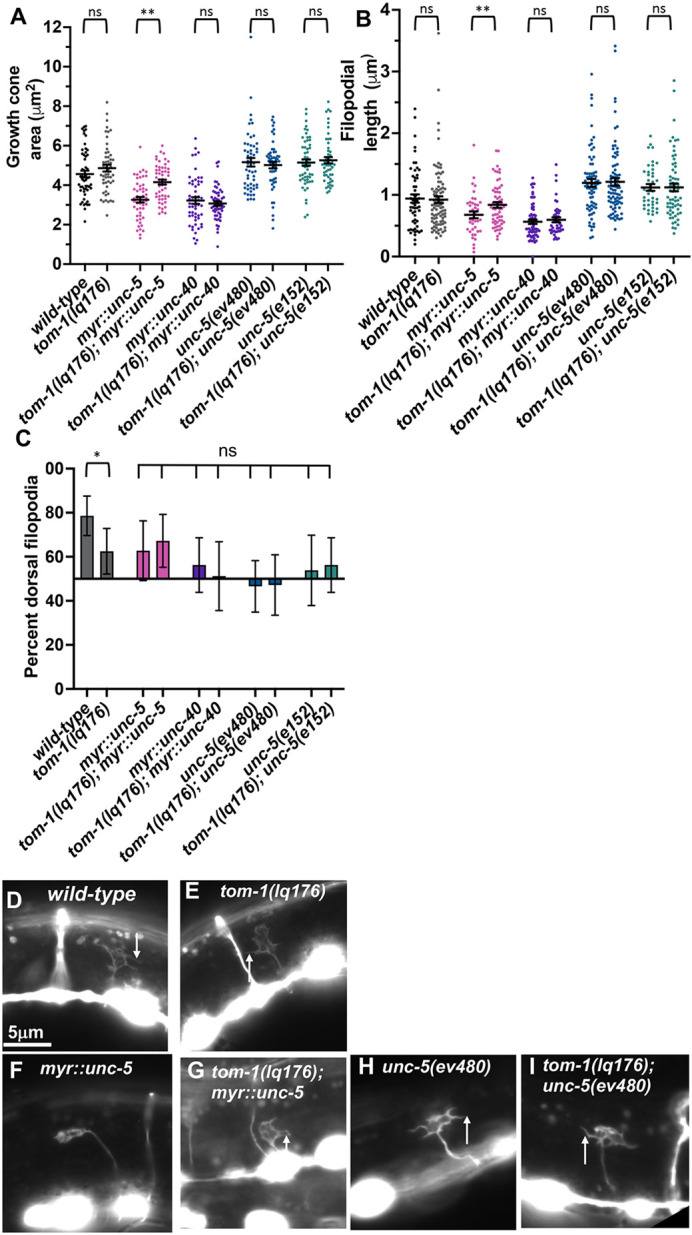
**Growth cone analysis of the short isoform-specific *tom-1(lq176)* mutant.** At least 50 growth cones of each genotype were analyzed. (A,B) Quantification of VD growth cone area and filopodial length as described in Fig. 4. Statistical comparisons between genotypes shown on other figures are not shown again here. Brackets indicate comparisons between genotypes. (C) Percentage of dorsally directed filopodial protrusions as described in Fig. 4. *tom-1(lq176)* mutants significantly perturbed growth cone polarity (brackets), but no *unc-5* double mutant showed any significant difference (ns, not significant). (D-I) Fluorescence micrographs of mutant VD growth cones of the indicated genotypes expressing *Punc-25::gfp*. Arrows point to filopodial protrusions. The image in D is a cropped version of the wild-type growth cone shown in Fig. 1D and is also shown in Figs 4D and 9D as a representative image of a wild-type growth cone. The image of the *myr::unc-5* growth cone in F is identical to the representative *myr::unc-5* growth cone image shown in Fig. 5D. Dorsal is up; anterior is left. Scale bar: 5 μm.

*tom-1(lq176)* did not affect growth cone area and filopodial length compared with wild type (Fig. 7A,B,D,E), but did display growth cone polarity defects (Fig. 7C). *tom-1(lq176)* suppressed the inhibition of growth cone area and filopodial length caused by *myr::unc-5* (Fig. 7A,B,F,G), but did not suppress the excessive growth cone protrusion of hypomorphic *unc-5(ev480 and e152)* mutants (Fig. 7A,B,H,I). No VD growth cones were apparent in *tom-1(lq176); unc-5(e791)* null double mutants. All double mutants with *tom-1(lq176)*, *unc-5* hypomorphs, and *myr::unc-5* displayed slightly increased VD/DD axon guidance defects, which were in some cases statistically significant (Fig. 3). Furthermore, VD growth cone polarity in double mutants resembled each single mutant alone, which all had unpolarized growth cones (Fig. 7C). *tom-1(lq176)* also displayed increased levels of axon guidance defects relative to wild type, but this was not statistically significant.

In sum, *tom-1(lq176)* resembled *tom-1(ok2437)* in all respects, including loss of growth cone polarity, suppression of growth cone inhibition by activated *myr::unc-5*, and failure to suppress excess growth cone protrusion in *unc-5* hypomorphic mutants. This indicates that the TOM-1 long isoforms alone cannot supply full TOM-1 function and is consistent with long and short isoforms having opposing roles (long are pro-protrusive; short is anti-protrusive).

### Transgenic expression of the TOM-1B short isoform (TOM-1S) inhibits VD growth cone protrusion

**Fig. 8.**
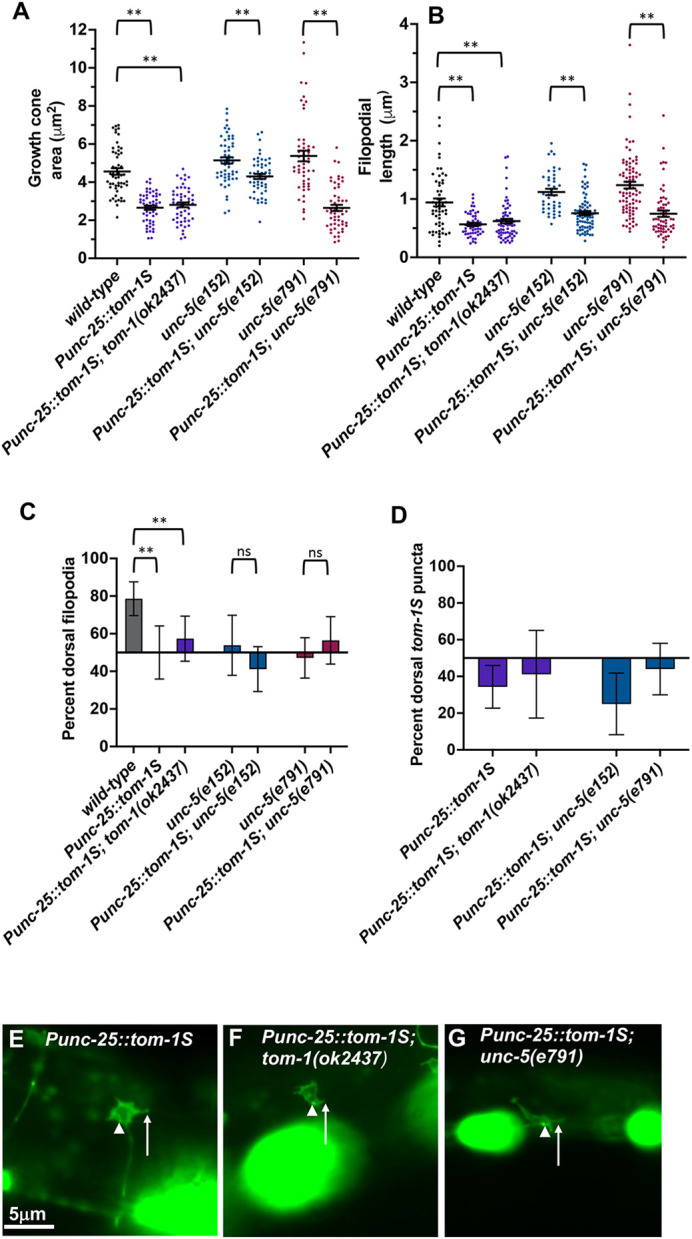
**Growth cone analysis of transgenic expression of *tom-1S.*** In this figure, *Punc-25::tom-1S* represents transgene TOM-1B short. At least 50 growth cones of each genotype were analyzed. (A,B) Quantification of VD growth cone area and filopodial length as described in Fig. 4. Statistical comparisons between genotypes shown on other figures are not shown again here. Brackets indicate comparisons between genotypes. (C) Percentage of dorsally directed filopodial protrusions as described in Materials and Methods. (D) Percentage of dorsally localized *tom-1S* GFP puncta filopodial protrusions as described in Fig. 4. (E-G) Fluorescence micrographs of mutant VD growth cones of the indicated genotypes. The GFP fluorescence is from the *Punc-25::tom-1S::gfp* transgene. Arrows point to filopodial protrusions and arrowheads point to the punctate GFP localization of TOM-1S. Dorsal is up; anterior is left. Scale bar: 5 μm.

The genomic region of the *tom-1S* short isoform was placed under the control of the *unc-25* promoter expressed in the VD/DD neurons. Transgenic expression of this construct in a wild-type background resulted in reduced VD growth cone area and filopodial length (Fig. 8A,B,E). Transgenic expression of the *tom-1S* short isoform also abolished VD growth cone polarity of protrusion (Fig. 8C). Similar results were seen with *tom-1(ok2437)*, an allele affecting both long and short isoforms (Fig. 5A,B,G). These data indicate that TOM-1S inhibits growth cone protrusion, consistent with the loss-of-function studies of *tom-1(lq176)*. These data also indicate that *tom-1S* can act cell-autonomously to inhibit growth cone protrusion. To investigate further whether TOM-1S isoform acts downstream of UNC-5, we analyzed double mutants of *Punc-25::tom-1S* short and *unc-5*. Double mutants displayed significant suppression of growth cone area and filopodial length compared with single *unc-5* mutants (Fig. 8A,B,G), confirming that the TOM-1S short isoform inhibits growth cone protrusion and acts downstream of UNC-5. No significant changes were seen in polarity of protrusion of double mutants of *unc-5* and the *tom-1S* isoform (Fig. 8C).

*Punc-25::tom-1S* was tagged with *gfp*, and fluorescence was present throughout the growth cones (Fig. 8D,E). TOM-1S::GFP-positive puncta were also observed in the growth cones. These puncta were significantly enriched in the ventral region of the growth cone (Fig. 8D,E). Similar localization of puncta was observed in *tom-1(ok2437)* and *unc-5(e791)* mutants (Fig. 8D,F,G). Transgenic expression of TOM-1S resulted in a significant increase in the VD/DD axon guidance defects (Figs 2O and 3A).

**Fig. 9.**
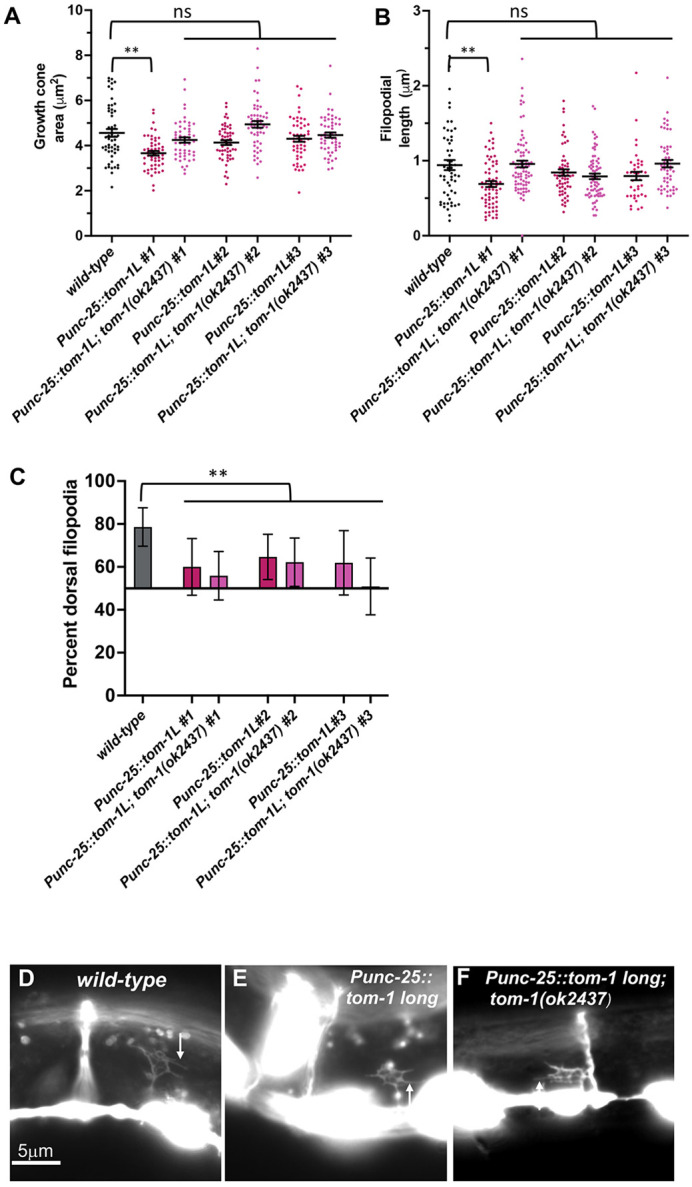
**Growth cone analysis of transgenic expression of *tom-1L.*** At least 50 growth cones of each genotype were analyzed. (A,B) Quantification of VD growth cone area and filopodial length as described in Fig. 4. Significance is compared with the wild type. (C) Percentage of dorsally directed filopodial protrusions as described in Fig. 4. (D-F) Fluorescence micrographs of mutant VD growth cones of the indicated genotypes. The image in D is a cropped version of the wild-type growth cone shown in Fig. 1D and is also shown in Figs 4D and 7D as a representative image of a wild-type growth cone. Arrows point to filopodial protrusions. Dorsal is up; anterior is left. Scale bar: 5 μm.

For transgenic expression of TOM-1L, cDNA of the *tom-1* long isoform A (TOM-1L), was placed under the control of the *unc-25* promoter expressed in the VD/DD neurons. Three independent transgenic lines were scored. Two showed no significant change to growth cone morphology, and one displayed a decrease in the growth cone area and filopodial length (Fig. 9A,B,D,E). There was no significant change in the *tom-1(ok2437)* background. All three lines displayed a significant decrease in the dorsal polarity of protrusions (Fig. 9C), and axon guidance defects (Fig. 3A). Transgenic expression of TOM-1L had no consistent effect on growth cone protrusion, but did perturb growth cone polarity. No GFP fluorescence was detected in TOM-1L::GFP transgenic lines, suggesting that it is expressed at very low levels.

In sum, transgenic expression of TOM-1S resulted in inhibition of growth cone protrusion, consistent with the loss-of-function studies. Transgenic expression of both TOM-1S and TOM-1L perturbed growth cone polarity. Finally, TOM-1S::GFP puncta localized to the ventral region of the growth cone, consistent with where TOM-1S might be active in inhibiting protrusion.

### *unc-64* regulates growth cone protrusion and genetically interacts with *unc-5*

Tomosyn inhibits vesicle fusion by interacting with the T-SNARE syntaxin via the tomosyn C-terminal R-SNARE domain, preventing syntaxin interaction with the vesicle SNARE VAMP (Fujita et al., 1998; Rizo and Rosenmund, 2008). *unc-64* encodes for the *C. elegans* homolog of syntaxin (Saifee et al., 1998). Complete loss of *unc-64* is lethal, but the hypomorphic *unc-64(md130)* allele is viable and displays slightly uncoordinated locomotion (Metz et al., 2007).

**Fig. 10.**
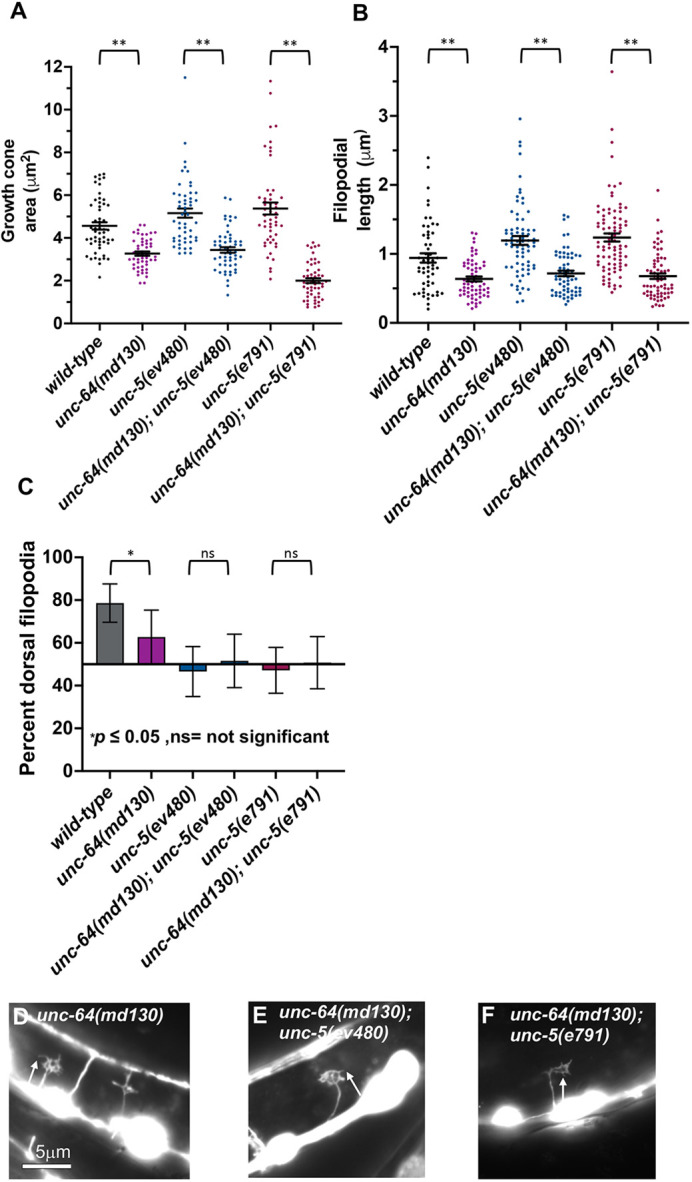
**Growth cone analysis in *unc-64* and *unc-5* mutants.** At least 50 growth cones of each genotype were analyzed. (A,B) Quantification of VD growth cone area and filopodial length as described in Fig. 4. (C) Percentage of dorsally directed filopodial protrusions in VD growth cones of different genotypes as described in Fig. 4. (D-F) Fluorescence micrographs of mutant VD growth cones expressing *Punc-25::gfp*. Arrows point to filopodial protrusions. Dorsal is up; anterior is left. Scale bar: 5 μm.

*unc-64(md130)* mutants displayed VD growth cones with reduced area and filopodial length compared with wild type (Fig. 10A,B,D). Furthermore, *unc-64(md130)* growth cones were unpolarized (Fig. 10C). Finally, *unc-64(md130)* suppressed excessive growth cone protrusion in *unc-5* null and hypomorphic mutants (Fig. 10A,B,E,F). *unc-64(md130)* displayed weak axon guidance defects alone, but double mutants of *unc-64; unc-5* displayed a synergistic enhancement in VD/DD axon guidance (Figs 2P and 3). *tom-1; unc-64* double mutants were lethal and could not be analyzed.

These data indicate that UNC-64 is required for VD/DD growth cone protrusion, including growth cone area and filopodial length. Loss of *unc-64* suppressed the large protrusive phenotype of *unc-5* mutants. This suggests that UNC-64 might be overactive in *unc-5* mutants, resulting in excessive growth cone protrusion in *unc-5* mutants.

## DISCUSSION

**Fig. 11.**
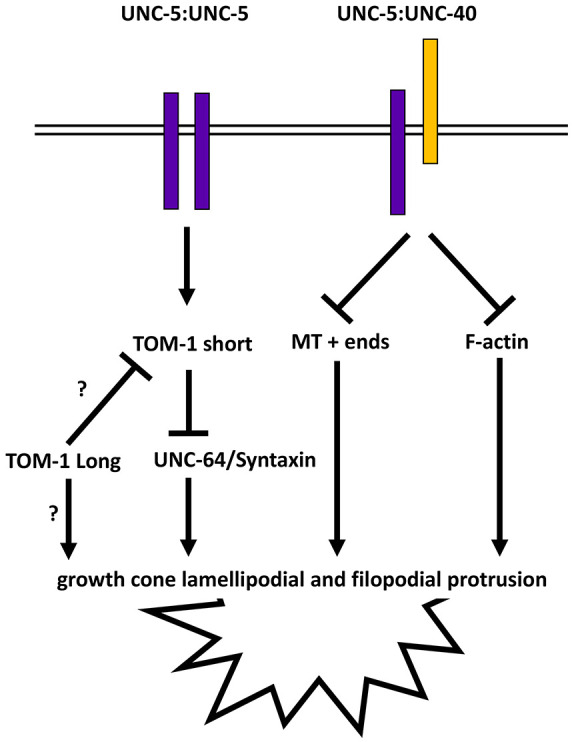
**Genetic model of TOM-1 inhibiting growth cone protrusions by preventing vesicle fusion.** Previous studies indicate that UNC-5 homodimers and UNC-5:UNC-40 heterodimers inhibit growth cone protrusion through the flavin monooxygenases (FMOs) and possible F-actin destabilization, and via UNC-33/CRMP and restriction of microtubule plus-end entry into growth cones. Studies here delineate a third pathway through which UNC-5 inhibits protrusion involving TOM-1 and the inhibition of vesicle fusion mediated by UNC-64. Our results indicate that a short isoform of TOM-1 containing only the V-SNARE domain is the active isoform in inhibiting protrusion and possible interaction with UNC-64/Syntaxin. TOM-1L isoform might act in opposition to TOM-1S and act in pro-protrusive manner. This might be a direct effect on protrusion, or possibly an auto-inhibitory effect on TOM-1S.

Previous studies have shown that in the polarity/protrusion model of growth cone outgrowth, UNC-6 inhibits VD growth cone lamellipodial and filopodial protrusion via the UNC-5 receptor. UNC-6 also polarizes the VD growth cone via UNC-5, biasing filopodial protrusion to the dorsal direction of outgrowth. By inhibiting protrusion ventrally and stimulating protrusion dorsally (via the UNC-40 receptor), UNC-6 directs dorsal migration of the VD growth cone. Previous studies showed that UNC-5 inhibits growth cone protrusion using FMOs, which might oxidize and destabilize F-actin in a similar manner to MICAL. UNC-5 also inhibits protrusion by restricting entry of microtubule plus-ends into growth cones, which have a pro-protrusive effect. Results here suggest that UNC-5 inhibits protrusion via a third pathway involving UNC-64 and the syntaxin inhibitor TOM-1 (Fig. 11) and that UNC-64 is required for growth cone protrusion, and TOM-1 is an inhibitor of growth cone protrusion. Given the known roles of syntaxin and tomosyn in regulating vesicle fusion, these results are consistent with the idea that UNC-5 prevents vesicle fusion in the growth cone through TOM-1 inhibition of UNC-64-mediated vesicle fusion. Prevention of vesicle fusion would restrict plasma membrane expansion and possibly delivery of pro-protrusive molecules (e.g. Arp2/3) and thus inhibit growth cone protrusion. *unc-64* and *tom-1* mutant VD growth cones were also unpolarized, indicating that regulation of vesicle fusion is required for establishing and/or maintaining growth cone polarity. Loss of *tom-1* had relatively mild effects on the growth cone and axon guidance that were revealed in sensitized backgrounds. This might reflect the role of vesicle fusion in regulating growth cone outgrowth and axon guidance in parallel to the previously described effects of actin and microtubules. Alternatively, there might be genes that act in parallel to *tom-1* in this process.

Vesicle exocytosis is essential for controlling growth cone membrane dynamics and extensions by fusion of plasmalemmal precursor vesicles at the plasma membrane of growth cones (Futerman and Banker, 1996). Studies on rat cultured hippocampal neurons suggest that exocytosis is restricted to the peripheral region of growth cones, which leads to membrane addition and extension, through the action of tomosyn in the growth cone ‘palm’ (Sakisaka et al., 2004). The *in vivo* results presented here are consistent with the idea that TOM-1 restricts vesicle fusion in the growth cone.

### *tom-1* encodes long and short isoforms with distinct functions

The *C. elegans* genome contains one tomosyn gene, *tom-1*, which encodes N-terminal WD40 repeats and a C-terminal R-SNARE domain, similar to tomosyn in other species. *tom-1* produces multiple isoforms, including a series of long isoforms containing the WD40 repeats and the R-SNARE domain, and a short isoform containing only the R-SNARE domain, produced by an alternative 5′ exon. This short isoform has not been described in *Drosophila* or vertebrates, but our results here suggest that it is an active isoform of TOM-1. Genetic results here indicate that TOM-1L isoforms have pro-protrusive roles in the growth cone, whereas TOM-1S inhibits protrusion. The short-isoform-specific *tom-1(lq176)* mutant resembled *tom-1(ok2437)*, suggesting that the long isoform cannot provide TOM-1 function in the absence of the short isoform. These data suggest that the TOM-1S isoform is important for inhibiting growth cone protrusion, possibly through interaction with UNC-64. Transgenic expression of TOM-1S resulted in smaller growth cones with shorter filopodia, consistent with this model. TOM-1L might have pro-protrusive roles independent of the short isoform. Alternately, TOM-1L might regulate TOM-1S and inhibit its function (Fig. 11).

### TOM-1S acts downstream of UNC-5 to inhibit the growth cone protrusion

Previous work showed that the UNC-6/Netrin receptors UNC-40 and UNC-5 regulate growth cone protrusion. UNC-40 stimulates protrusion whereas UNC-5 inhibits protrusion, and asymmetric distribution of protrusive activity across the growth cone results in directed growth cone migration away from UNC-6/Netrin (the Polarity/Protrusion model) (Gujar et al., 2018). We also showed that UNC-5 inhibits protrusion via the FMOs by possible actin destabilization (Gujar et al., 2017), and by preventing MT entry via UNC-33/CRMP (Gujar et al., 2017; Gujar et al., 2019).

Here we show that the TOM-1S isoform is required for UNC-5 to inhibit VD growth cone protrusion. The *tom-1(ok2437)* an allele affecting both the short and long isoforms and the short-isoform-specific *tom-1(lq176)* both suppressed the inhibitory effect of MYR::UNC-5 on VD growth cone protrusion. Although neither mutant alone had significantly increased growth protrusion expected of an inhibitory molecule, the effects might be masked by the other pathways downstream of UNC-5 (FMOs and UNC-33/CRMP), or by another redundant pathway. MYR::UNC-5 is a sensitized genetic background that revealed the effects on *tom-1* mutants on the VD growth cone. *tom-1(ok2437)* and *tom-1(lq176)* did not suppress the inhibitory effects on MYR::UNC-40, suggesting that TOM-1 might act specifically downstream of UNC-5 homodimers and not UNC-5:UNC-40 heterodimers. Finally, *tom-1(ok2437)* and *tom-1(lq176)* did not affect the large, overly protrusive growth cones of *unc-5* loss-of-function mutants, consistent with a role in inhibiting protrusion.

### TOM-1L have a pro-protrusive role in the VD growth cone

In contrast to the *tom-1(ok2437)* which affects both long and short isoforms and *tom-1(lq176)* short isoform specific mutant, the long isoform-specific mutant *tom-1(nu468)* displayed VD growth cones with reduced area and filopodial length. *tom-1(nu468)* was required for the large, overly protrusive growth cones in *unc-5* null mutants, and did not suppress MYR::UNC-5. Together, these data indicate that TOM-1 long isoforms have a pro-protrusive role in the growth cone, the opposite of TOM-1 short. TOM-1L isoforms were required for excess protrusion in *unc-5* mutants, suggesting that, in the absence of UNC-5, TOM-1L is overactive.

Previous neurophysiological studies indicate that the TOM-1 long isoforms are inhibitory to neurosecretion (Dybbs et al., 2005; Gracheva et al., 2006; McEwen et al., 2006; Gracheva et al., 2010; Burdina et al., 2011). Expression of the R-SNARE domain alone has been shown to be insufficient to restore inhibition of synaptic transmission (Burdina et al., 2011), whereas experiments here show that expression of the TOM-1 short isoform inhibited VD growth cone protrusion. Possibly, the function of the long and short isoforms in vesicle fusion are cell and context specific. Indeed, in cultured superior cervical ganglion neurons, tomosyn RNAi inhibited the evoked response (Baba et al., 2005), the opposite of what is expected of an inhibitor of vesicle fusion. In the VD growth cone, TOM-1L might act as a true ‘friend to syntaxin’, possibly inhibiting the function of TOM-1 short. It is also possible that TOM-1 long isoforms have a syntaxin-independent stimulatory effect on growth cone protrusion.

### TOM-1L and S isoforms are both required for VD growth cone polarity and VD/DD axon guidance

All *tom-1* mutants analyzed here displayed loss of dorsally polarized filopodial protrusions on the growth cone. Transgenic expression of the TOM-1S also resulted in VD growth cone polarity defects. This indicates that both TOM-1L and TOM-1S are required to establish and/or maintain VD growth cone polarity in a complex and likely dynamic manner. No genetic interaction analyzed here modified the polarity defect, so it is impossible to say whether TOM-1 acts downstream of UNC-5 in growth cone polarity.

All *tom-1* alleles displayed increased VD/DD axon guidance defects (but this was not statistically significant compared with wild type), and all of them synergistically enhanced the VD/DD axon guidance defects in double mutant combinations with *unc-5*. This suggests that both isoforms of *tom-1* are necessary to maintain proper axon guidance and again supports the hypothesis that TOM-1 acts downstream of UNC-5 signaling.

### UNC-64 is required for robust VD growth cone protrusion and polarity

Tomosyn is very well-characterized as an inhibitor of vesicle fusion by blocking interaction of syntaxin with the V-SNARE synaptobrevin, including *C. elegans* TOM-1 in neurosecretion (Dybbs et al., 2005; Gracheva et al., 2006; McEwen et al., 2006; Gracheva et al., 2010; Burdina et al., 2011). It is possible that the effects of TOM-1 in growth cone protrusion are independent of vesicle fusion. However, hypomorphic *unc-64* mutants displayed reduced VD growth cone area, shorter filopodial protrusions, and a loss of polarity of filopodial protrusions. This is similar to the long isoform-specific *tom-1(nu468)* mutant and is consistent with a role of UNC-64/syntaxin in promoting growth cone protrusion and polarity. This strongly indicates that vesicle fusion is required for robust VD growth cone protrusion, filopodial protrusion, and polarity of filopodial protrusion. The effects of TOM-1 on these processes are thus likely to be mediated through regulation of vesicle fusion.

### The UNC-6 receptor UNC-5 inhibits VD growth cone protrusion via TOM-1

In cultured rat hippocampal neurons, tomosyn prevents vesicle fusion at the ‘palm’ of the growth cone, directing vesicle fusion to the extending growth cone tip (Sakisaka et al., 2004). Evidence is presented here *in vivo* in *C. elegans* that TOM-1 might act similarly in the VD growth cone. Localization of TOM-1S puncta to the ventral region of the VD growth cone suggests that TOM-1S might be active at the base of the VD growth cone. Loss of UNC-64 also resulted reduced growth cone protrusion. We speculate that TOM-1S prevents vesicle fusion ventrally, resulting in inhibited ventral growth cone protrusion.

We show that TOM-1 acts downstream of the UNC-6 receptor UNC-5 to inhibit protrusion. However, TOM-1S localization ventrally was not dependent on UNC-5A, suggesting that UNC-5 might activate TOM-1S and some other mechanism results in ventral localization of TOM-1S. Given that UNC-5 also polarizes the growth cone, the activity of TOM-1 ventrally and laterally might be controlled by UNC-5. TOM-1 is also required to establish and/or maintain growth cone polarity of protrusion, suggesting the role of vesicle fusion in this process as well. In cultured rat hippocampal neurons, upon growth cone collapse, tomosyn extends throughout the growth cone. This situation might be analogous to constitutively active MYR::UNC-5, which might constitutively recruit TOM-1 throughout the entire growth cone, leading to inhibited protrusion. These results advance our understanding of the role of the UNC-6 receptor UNC-5 in growth cone morphology during outgrowth. They show that, in addition to the two pathways involving F-actin and microtubule plus-end entry, UNC-5 inhibits protrusion by preventing vesicle fusion in the growth cone using TOM-1.

## MATERIALS AND METHODS

### Genetic methods

Experiments were performed at 20°C using standard *C. elegans* techniques (Brenner, 1974). Mutations used were: LGI: *tom-1(ok2437, nu468, lq176), wrdSi23*. LGII: *juIs76* [*Punc-25*::*gfp*]. LGIII: *unc-64(md130)*. LGIV: *unc-5(e791, ev480, e152)*. The presence of mutations was confirmed by phenotype and sequencing. Chromosomal locations not determined: *lqIs296[Punc-25::myr::unc-5::gfp], lqIs128[Punc-25::myr::unc-40::gfp], lqIs383 [Punc-25::tom-1S::gfp], lqIs346, lqIs347, lqIs382 [Punc-25::tom-1L::gfp #1,2,3]*.

### Transgene construction

*Punc-25::tom-1S* (pEL1154; supplementary file S1) was made by amplifying the entire genomic region of the *tom-1B* short isoform by PCR, and placing this downstream of the *unc-25* promoter to drive expression in the VD/DD neurons.

*Punc-25::tom-1L* (pEL1147; supplementary file S2) was made by amplifying the cDNA of *tom-1A* from mixed-stage N2 RNA using the SuperScript^TM^ IV one-step RT-PCR kit (Invitrogen) and placed downstream of the *unc-25* promoter to drive expression in the VD/DD neurons. The coding regions for both transgenes were sequenced to ensure no errors had been introduced by PCR.

### Cas9 genome editing to generate *tom-1B(lq176)*

CRISPR-Cas9 genome editing was used to delete in a precise manner the entire intron 18 of *tom-1A*, between exons 18 and 19 (Fig. 1). This removes the first exon of the *tom-1B* short isoform (*tom-1S*), which resides in intron 18, and leaves the coding potential of *tom-1* long isoforms unchanged. Synthetic guide RNAs were directed at the 5′ and 3′ ends of the intron: sgRNA1 against *tom-1* short: 5′ CATCAATTTCCACAGAATGT 3′; sgRNA2 against *tom-1* short: 5′ TTACATGGCAAGTCAAACAG 3′.

A mix of sgRNAs, a single-stranded oligonucleotide repair template, and Cas9 enzyme was injected into the gonads of N2 animals, along with the *dpy-10(cn64)* co-CRISPR reagents (El Mouridi et al., 2017). Deletion of *tom-1* intron 18 was confirmed by PCR and sequencing. A single-stranded oligodeoxynucleotide was used as a repair template, which recoded the sgRNA region to maintain the same coding potential. The recoded sequence of *lq176* is: TGAGAATTCTTCAAACTTCTACGATTTTCCCACACTCCGTCGAGATCGACGACCCACTCTGCCAAAAGACCGCCTTCTCCGACCATGGACTCGGAGTCTATATGGCCTCCCAAACAGAGGTAAGATACTTTGTTTTATCATGAAAGTTA. The mutation was named *tom-1(lq176)*. Genome-editing reagents were produced by InVivo Biosystems.

### RNA-seq and analysis

Total RNA was isolated from three independent isolates of mixed-stage animals of the strain LE6194 (SSM1, SSM2 and SSM3) as previously described (Tamayo et al., 2013). The LE6194 genotype is *wrdSi23 I; juIs76 II* and is wild type for the *unc-5* gene. Stranded poly-A RNA-seq libraries were constructed using the NEBNext^®^ Ultra™ II Directional RNA Library Prep Kit for Illumina and subjected to paired-end 150 cycle sequencing on a Nextseq550. Reads were aligned to the *C. elegans* genome using HISAT2 with default settings (version 2.1.0) (Kim et al., 2019). The resulting BAM files were analyzed in the Integrated Genome Viewer (Robinson et al., 2011; Thorvaldsdottir et al., 2012) from which the Sashimi plot for SSM1 in Fig. 1B was generated. The following numbers of paired reads for each sample mapped to the genome: SSM1, 46,067,340; SSM2, 52,240,104; SSM3, 55,063,010.

### Statistics

Proportional data were analyzed using Fisher's Exact test, and continuous data with unpaired, two-tailed Student's *t*-test with unequal variance. With an α false-positive value of 0.05, sample sizes used each gave an estimated statistical power of >80% (a β false-negative value of <0.2).

### Quantification of axon guidance defects

VD/DD neurons were visualized with the *Punc-25*::*gfp* transgene *juIs76* (Jin et al., 1999), which is expressed in GABAergic motor neurons including 13VDs and 6DDs. Axon guidance defects were scored as previously described (Mahadik and Lundquist, 2022). In wild type, an average of 16 of the 19 commissures of VD/DD axons are distinguishable, as axons can be present in a fascicle and thus cannot be resolved. A total of 100 animals were scored (1600 total commissural processes). At a false-positive α value of 0.05 with observed variances of 5% or greater, scoring 1600 axons gave an estimated statistical power of ≥80%. Total axon guidance defects were calculated by counting all the axons that failed to reach the dorsal nerve cord, wandered at an angle of 45° or greater, crossed over other processes, and displayed ectopic branching. Significance difference between two genotypes was determined by using Fisher's exact test.

### Growth cone imaging and quantification

VD growth cones were imaged as previously described (Norris and Lundquist, 2011; Norris et al., 2014; Gujar et al., 2017, 2018, 2019; Mahadik and Lundquist, 2022). Late L1/early L2 larval animals were harvested 16-h post-hatching at 20°C and placed on a 2% agarose pad with 5 mM sodium azide in M9 buffer. Compared with wild type, mutants sometimes displayed delayed emergence of VD/DD growth cones from the ventral nerve cord, so growth cones were imaged when they emerged. At *n*=50, at a false-positive α value of 0.05, statistical power of β (false negative) was much lower than 0.2.

Growth cones were imaged with a Qimaging Retiga EXi camera on a Leica DM5500 microscope at 100× magnification. Projections<0.5 μm in width were scored as filopodia. Growth cone area and filopodial length were quantified using ImageJ software. Quantification was performed as described previously (Norris and Lundquist, 2011; Norris et al., 2014; Gujar et al., 2017, 2018, 2019; Mahadik and Lundquist, 2022). Significance of difference between two genotypes was determined by two-sided *t*-test with unequal variance.

Polarity of growth filopodial protrusions was determined as previously described (Norris and Lundquist, 2011; Norris et al., 2014; Gujar et al., 2017, 2018, 2019; Mahadik and Lundquist, 2022). Growth cone images were divided into two halves, dorsal and ventral, with respect to the ventral nerve cord. The number of filopodia in each half was counted. The proportion of dorsal filopodia was calculated as the number of dorsal filopodia divided by the total number of filopodia. Significance of difference between two genotypes was determined by Fisher's exact test.

## Supplementary Material

10.1242/develop.201031_sup1 Supplementary information
