# Supplementary material for: TOM-1/tomosyn acts with the UNC-6/netrin receptor UNC-5 to inhibit growth cone protrusion in Caenorhabditis elegans
Source: Development. 2023 Apr 4;150(7):dev201031. doi: 10.1242/dev.201031 (PMC10112904; doi:10.1242/dev.201031)
Supplement: Supplementary information [file develop-150-201031-s1.pdf]

File S1. *Punc-25::tom-1S* sequence.

Click here to download File 1

File S2. *Punc-25::tom-1L* sequence.

Click here to download File 2

Development • Supplementary information
